# Glucose-Mediated Protein Arginine Phosphorylation/Dephosphorylation Regulates *ylxR* Encoding Nucleoid-Associated Protein and Cell Growth in *Bacillus subtilis*

**DOI:** 10.3389/fmicb.2020.590828

**Published:** 2020-09-25

**Authors:** Mitsuo Ogura

**Affiliations:** Institute of Oceanic Research and Development, Tokai University, Shizuoka, Japan

**Keywords:** glucose response, YwlE phosphatase, ClpCP protease, protein acetylation, glycolysis

## Abstract

Glucose is the most favorable carbon source for many bacteria, and these bacteria have several glucose-responsive networks. We proposed new glucose responsive system, which includes protein acetylation and probable translation control through TsaEBD, which is a tRNA modification enzyme required for the synthesis of threonylcarbamoyl adenosine (t^6^A)-tRNA. The system also includes nucleoid-associated protein YlxR, regulating more than 400 genes including many metabolic genes and the *ylxR*-containing operon driven by the P*ylxS* promoter is induced by glucose. Thus, transposon mutagenesis was performed for searching regulatory factors for P*ylxS* expression. As a result, *ywlE* was identified. The McsB kinase phosphorylates arginine (Arg) residues of proteins and the YwlE phosphatase counteracts against McsB through Arg-dephosphorylation. Phosphorylated Arg has been known to function as a tag for ClpCP-dependent protein degradation. The previous analysis identified TsaD as an Arg-phosphorylated protein. Our results showed that the McsB/YwlE system regulates P*ylxS* expression through ClpCP-mediated protein degradation of TsaD. In addition, we observed that glucose induced *ywlE* expression and repressed *mcsB* expression. It was concluded that these phenomena would cause glucose induction (GI) of P*ylxS*, based on the Western blot analyses of TsaD-FLAG. These observations and the previous those that many glycolytic enzymes are Arg-phosphorylated suggested that the McsB/YwlE system might be involved in cell growth in glucose-containing medium. We observed that the disruption of *mcsB* and *ywlE* resulted in an increase of cell mass and delayed growth, respectively, in semi-synthetic medium. These results provide us broader insights to the physiological roles of the McsB/YwlE system and protein Arg-phosphorylation.

## Introduction

Glucose is the most favorable carbon source for many bacteria, so bacteria have developed several glucose-responsive networks (Deutscher, 2008). In Gram-positive bacteria, such as *Bacillus subtilis*, the transcription factor, catabolite control protein A (CcpA), is the master regulator for carbon catabolite regulation (Deutscher, 2008; Fujita, 2009). Incorporating glucose into the bacterial cells increases the metabolite, fructose-1,6-bisphosphate, which triggers HPr phosphorylation at Ser46. HPr is a phosphocarrier protein in the sugar phosphotransferase system and P-Ser-HPr activates CcpA, causing widespread transcriptional changes. Moreover, there are several additional glucose-responsive transcription factors, such as CcpC, CcpN, CggR, and GlcT (Fujita, 2009).

**FIGURE 1:**
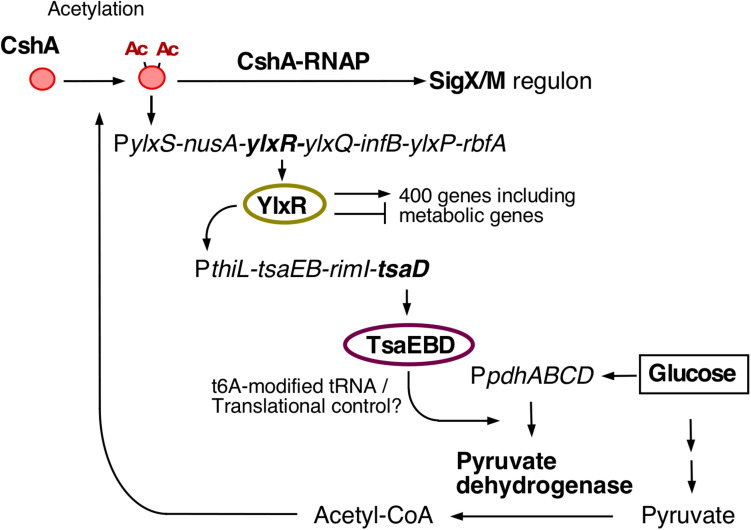
The current model for glucose-responsive system (GRS). The indicated pathways were previously identified: *in vivo* association of CshA with RNAP (Delumeau et al., 2011), glucose-stimulated CshA acetylation and *sigX* regulation (Kosono et al., 2015; Ogura and Asai, 2016), CshA-dependent P*ylxS* regulation driving YlxR expression, which regulates metabolic genes (Ogura and Kanesaki, 2018), and transcriptional regulation of *tsaEBD* through the functional YlxR-binding to the promoter of *tsaEBD*, whose products are assembled and regulate pyruvate dehydrogenase translation (Ogura et al., 2019). Pyruvate dehydrogenase provides acetyl-CoA, which would be the acetyl moiety source for CshA acetylation. In the *tsaD* disruptant grown with glucose cellular acetyl-CoA pool was reduced (Ogura et al., 2019). Each pathway is supported by experimental evidence, however, considering the whole regulatory cascade there is a room for further verification. Arrows indicate transcription, translation, acetylation, enzymatic reaction, transcriptional activation or metabolic reaction. T-bars indicate transcriptional repression. Ac, acetyl moiety; RNAP, RNA polymerase.

Several lines of evidence suggest another glucose responsive system (GRS) which includes protein acetylation and probable translational control (Figure 1; Ogura and Asai, 2016; Ogura and Kanesaki, 2018; Ogura et al., 2019, 2020). Glucose addition to culture medium often induces protein acetylation in *E. coli* and *B. subtilis* (Kosono et al., 2015; Schilling et al., 2015). Proteomic analysis of *B. subtilis* revealed that CshA, a DEAD-box helicase, is acetylated (Lehnik-Habrink et al., 2013; Kosono et al., 2015). We recently found that glucose stimulates CshA lysine acetylation (Ogura and Asai, 2016) and CshA associates with RNA polymerase (RNAP) (Delumeau et al., 2011). The association between acetylated CshA and RNAP enhances its SigX affinity, leading to glucose induction (GI) of *sigX* (Shiwa et al., 2015; Helmann, 2016; Ogura and Asai, 2016). GI of *sigX* caused by CshA acetylation is susceptible to pyruvate dehydrogenase (PDH) mutations in *pdhABCD* (Gao et al., 2002; Ogura and Asai, 2016). *pdh* gene disruption would reduce the intracellular acetyl-CoA pool and flux resulting from loss of PDH activity, that is, pyruvate conversion to acetyl-CoA (Gao et al., 2002). *ylxR*, a regulator of GRS, is another gene subject to GI caused by CshA (Ogura and Kanesaki, 2018). YlxR has characteristics specific to nucleoid-associated proteins (NAPs) and regulates the transcription of more than 400 genes (Dillon and Dorman, 2010; Ogura and Kanesaki, 2018). Further, YlxR is involved in *tsaEBD*-containing operon expression (Ogura et al., 2019). TsaEBD is a tRNA modification enzyme that is required for the synthesis of threonylcarbamoyl adenosine (t^6^A) (Thiaville et al., 2015; Zhang et al., 2015). t^6^A-modified tRNA is conserved in three domains of life and its deficiency sometimes causes severe dysfunctions (Thiaville et al., 2016; Arrondel et al., 2019; Ogura et al., 2019). In *B. subtilis*, several lines of evidence suggest a relationship between low t^6^A and protein quality control, including PDH (Ogura et al., 2019). Thus, t^6^A is required for stable acetyl-CoA supply through control of PDH activity. In other words, GRS constitutes feedback regulatory networks (Ogura et al., 2020).

Several bacterial protein modifications include Lys-acetylation, His-Asp phosphorylation for signal transduction by two-component regulatory systems, and Ser/Thr/Tyr phosphorylation (Dworkin, 2015; Mijakovic et al., 2016; Carabetta and Cristea, 2017). Emerging evidence suggests that Arg-phosphorylation is another protein modification in bacteria (Mijakovic et al., 2016). This modification was first identified in the transcriptional repressor CtsR-involving heat-shock response, and is regulated by McsA, McsB kinases, and YwlE phosphatase. Inactivated CtsR is degraded by ClpCP ATP-dependent protease (Krueger et al., 2001; Fuhrmann et al., 2009, 2013; Elsholz et al., 2012). Later, Arg-phosphorylation was identified as a tag for protein degradation by ClpCP (Trentini et al., 2016). Several efforts to identify Arg-phosphorylated proteins in *B. subtilis* and *Staphylococcus aureus* revealed many targets, including metabolic enzymes, translation-related proteins, and some transcription factors (Elsholz et al., 2012; Schmidt et al., 2014; Trentini et al., 2014; Junker et al., 2019).

Here, we report that *ywlE* is a regulatory factor for *ylxR* expression, which is driven by P*ylxS*. Previous analysis identified TsaD as an Arg-phosphorylated protein (Trentini et al., 2014). McsB kinase and YwlE phosphatase regulate P*ylxS* via ClpCP-mediated protein degradation of TsaD. In addition, we observed that glucose represses *mcsB* and *clpC* expression (Ishii et al., 2013) and induces *ywlE* expression. Many glycolytic and TCA cycle enzymes are Arg-phosphorylated, suggesting that the McsB/YwlE system might be involved in cell growth in glucose-containing medium (Elsholz et al., 2012; Schmidt et al., 2014; Trentini et al., 2014; Junker et al., 2019; Zhou et al., 2019). *mcsB* and *ywlE* disruption resulted in increased cell mass and delayed growth, respectively. These results provide broad insights to the physiological roles of the McsB/YwlE system and protein Arg-phosphorylation.

## Materials and Methods

### Strains, Media, and PCR

All *B. subtilis* strains used in this study are shown in Table 1 and Supplementary Figure 1. One-step competence medium (MC) (Kunst et al., 1994), Schaeffer’s sporulation medium (SM) (Schaeffer et al., 1965), Luria-Bertani (LB) medium (Lennox, Difco, MI, United States), and Antibiotic medium 3 (Difco) were used. Antibiotic concentrations were described previously (Ogura and Tanaka, 1996; Ogura et al., 1997). Synthetic oligonucleotides were commercially prepared by Tsukuba Oligo Service (Ibaraki, Japan) and are listed in Supplementary Table 1. For PCR-mediated construction of strains and plasmids, PrimeSTAR MAX DNA polymerase (Takara Co., Shiga, Japan) was used. For screening of recombinant DNA during plasmid construction, LA PCR DNA polymerase (Takara Co.) was used.

**TABLE 1:** Strains and plasmids used in this study.

**Strain**	**Genotype**	**Reference or source**
168	*trpC2*	Laboratory stock
YacId	*trpC2 yacI* (*mcsB* Em^r^ *lacZ*)	BSORF
OAM879	*trpC2 yacI* (*mcsB* Em^r^ *lacZ*::Tc^r^)	This study
OAM880	*trpC2 ywlE*::Tn (inserted to 127th codon of *ywlE* ORF, Km^r^)	This study
OAM881	*trpC2 ywlE*::Tc^r^	This study
OAM734	*trpC2 tsaD*::Tn (Km^r^)	Ogura et al., 2019
OAM588	*trpC2 clpC* (Tc^r^)	Ogura et al., 2003
OAM741	*trpC2 thrC*::P*ylxS-lacZ* (−284/+77^1^ Sp^r^)	Ogura and Kanesaki, 2018
OAM882	*trpC2 thrC*::P*ylxS-lacZ* (−284/+77^1^ Sp^r^) *tsaD*::Tn (Km^r^)	This study
OAM883	*trpC2 thrC*::P*ylxS-lacZ* (−284/+77^1^ Sp^r^) *ywlE*::Tn (Km^r^)	This study
OAM884	*trpC2 thrC*::P*ylxS-lacZ* (−284/+77^1^ Sp^r^) *yacI* (*mcsB* Em^r^ *lacZ*::Tc^r^)	This study
OAM945	*trpC2 thrC*::P*ylxS-lacZ* (−284/+77^1^ Sp^r^) *yacI* (*mcsB* Em^r^ *lacZ*::Tc^r^) *ywlE*::Tn (Km^r^)	This study
OAM885	*trpC2 thrC*::P*ylxS-lacZ* (−284/+77^1^ Sp^r^) *ywlE*::Tn (Km^r^) *amyE*::P*xyl-ywlE* (Cm^r^)	This study
OAM886	*trpC2 thrC*::P*ylxS-lacZ* (−284/+77^1^ Sp^r^) *tsaD*::Tn (Km^r^) *amyE*::P*xyl-tsaD* (Cm^r^)	This study
OAM946	*trpC2 thrC*::P*ylxS-lacZ* (−284/+77^1^ Sp^r^) *tsaD*::Tn (Km^r^) *amyE*::P*xyl-tsaD* (Cm^r^) *ywlE*::Tc^r^	This study
OAM887	*trpC2 thrC*::P*ylxS-lacZ* (−284/+77^1^ Sp^r^) *tsaD*::Tn (Km^r^) *amyE*::P*xyl-tsaD* (Cm^r^ R282K)	This study
OAM947	*trpC2 thrC*::P*ylxS-lacZ* (−284/+77^1^ Sp^r^) *tsaD*::Tn (Km^r^) *amyE*::P*xyl-tsaD* (Cm^r^ R282K) *ywlE*::Tc^r^	This study
OAM888	*trpC2* P*ywlE-lacZ* (Em^r^)	This study
RIK60	*trpC2 lys1 aprE nprR nprE amyE*::P*ctsR-bgaB* (Cm^r^)	Nanamiya et al., 1998
OAM891	*trpC2 amyE*::P*ctsR-bgaB* (Cm^r^)	This study
OAM893	*trpC2 ywlE-flag* (Cm^r^)	This study
OAM897	*trpC2 tsaD-flag* (Cm^r^)	This study
OAM898	*trpC2 tsaD-flag* (Cm^r^) *ywlE*::Tn (Km^r^)	This study
OAM899	*trpC2 tsaD-flag* (Cm^r^) *yacI* (*mcsB* Em^r^ *lacZ*::Tc^r^)	This study
QPB418	*clpC* (Tc^r^) PY79 background	Pan et al., 2001
OAM900	*trpC2 tsaD-flag* (Cm^r^) *clpC* (Tc^r^)	This study
OAM901	*trpC2 tsaD-flag* (Cm^r^) P*ywlE-lacZ* (Em^r^)	This study
OAM909	*trpC2 amyE*::P*thiL*-*tsaD-flag* (Cm^r^)	This study
OAM910	*trpC2 amyE*::P*thiL*-*tsaD-flag* (Cm^r^, R282K)	This study
OAM779	*trpC2 pdhD*::pMUT-His*-pdhD* (Em^r^)	Ogura et al., 2019
OAM908	*trpC2 pdhD*::pMUT-His*-pdhD* (Em^r^) *yacI* (*mcsB* Em^r^ *lacZ*::Tc^r^)	This study
Plasmid	Description	
pMarA	Amp^r^ Em^r^ Km^r^	Le Breton et al., 2006
pX	Amp^r^ *amyE*::*xylR*-P*xyl* Cm^r^	Hori et al., 2002
pX-ywlE	Px carrying *ywlE* (*ywlE* ORF with its SD), Cm^r^	This study
pX-tsaD	Px carrying *tsaD* (*tsaD* ORF with its SD), Cm^r^	Ogura et al., 2019
pX-tsaD-m	Px carrying *tsaD* (*tsaD* ORF with its SD and R282K mutation), Cm^r^	This study
pBEST304	Amp^r^ Tc^r^	Itaya, 1992
pMutinIII	Insertion vecter, ampicillin and erythromycin resistance, *lacZI*	Vagner et al., 1998
pMutin-PywlE	pMutinIII carrying PywlE	This study
pCA3xFLAG	Ampicillin resistance, FLAG, Cm^r^	Yamamoto et al., 2003
pywlE-flag	pCA3xFLAG carrying C-terminal region of *ywlE*	This study
ptsaD-flag	pCA3xFLAG carrying C-terminal region of *tsaD*	This study
pLacZ:Tc	Amp^r^ *lacZ*::Tc^r^	Ogura et al., 2003

*^1^Numbers indicate the nucleotide positions relative to the transcription start point.*

### Strain Construction

The *ywlE*::Tc^r^ unit in OAM881 was constructed using PCR. Briefly, Tc^r^ from pBEST304 (Itaya, 1992) and the upstream and downstream *ywlE* regions with overlapping Tc^r^ regions were amplified using primers listed in Supplementary Table 1, and then combined by PCR. The unit was directly transformed into *B. subtilis* 168. Total DNA was taken using DNeasy kit (Qiagen, Venlo, Netherlands) from the resultant Tc^r^ strain for PCR-based confirmation of the expected chromosomal structure. The wild and mutant strains bearing tag-added *tsaD* at the *amyE* locus (OAM909 and OAM 910) were constructed by the PCR-based method shown in Supplementary Figure 1. The ORF associated with its own promoter was sequenced.

### Plasmid Construction

The plasmids used in this study are listed in Table 1. For PCR, *B. subtilis* 168 chromosomal DNA was used as template. To construct pMutin-PywlE, PCR products were amplified using the oligonucleotides pair pMut-PywlE-F(H)/pMut-PywlE-R(B), digested with *Hin*dIII/*Bam*HI, and cloned into pMutin3 treated with the same enzymes (Vagner et al., 1998). To construct pX-ywlE, PCR products were amplified using the oligonucleotides pair pX-ywlE-Spe/pX-ywlE-Bam, digested with *Spe*I/*Bam*HI, and cloned into a pX plasmid treated with the same enzymes (Hori et al., 2002). To construct pX-tsaD-m, PCR products were amplified using the oligonucleotide pairs pX-gcp-Spe/tsaD-M1 and pX-gcp-Bam/tsaD-M2. Both PCR products were combined by PCR using the oligonucleotide pair pX-gcp-Spe/pX-gcp-Bam. The final PCR product was digested with *Spe*I/*Bam*HI and cloned into pX treated with the same enzymes. To construct pywlE-flag and ptsaD-flag, PCR products were amplified using the oligonucleotides pairs Pflag-ywlE-F-H/Pflag-ywlE-R-Xb and Pflag-tsaD-F-H/Pflag-tsaD-R-Xb and digested with *Hin*dIII/*Xba*I. Each product was cloned into pCA3xFLAG treated with the same enzymes (Yamamoto et al., 2003).

### Transposon Mutagenesis

The transposon delivery vector pMarA was introduced into the strain OAM741 (Le Breton et al., 2006). The resultant strain was incubated in LB medium containing kanamycin at 30°C overnight. The cells were diluted and plated onto sporulation medium with 1.5% agar plates containing X-gal (100 μg/mL), kanamycin, spectinomycin and 2% glucose. The plates were incubated at 42°C. White colonies were selected. The insertion mutations were backcrossed into the parental strain and used with the Lac assay. Total DNA was isolated from the candidate strain, *Sau*IIIA1-digested, ligated, and amplified with inverse PCR using oligonucleotides 695 and 696, as described previously (Chan et al., 2014; Ogura and Asai, 2016; Supplementary Table 1). The PCR products were sequenced using the oligonucleotide 696.

### β-Galactosidase Analysis

Growth conditions and β-galactosidase analysis procedures were previously described (Ogura and Asai, 2016; Ogura and Kanesaki, 2018). β-galactosidase activity from BgaB was determined at 54°C. β-galactosidase analysis using chlorophenol red β-D-galactopyranoside (CPRG, Roche, Germany) was performed as described previously (Ogura et al., 2019).

### Western Blot Analysis

To determine the amounts of each FLAG protein and SigA, cells were grown in 50 mL sporulation medium with or without 2% glucose in 200 mL flasks. At the appropriate growth phase, cells were harvested and washed with 1 mL TBS buffer (10 mM Tris-HCl pH 7.5 and 150 mM NaCl) containing 1 mM phenylmethylsulfonyl fluoride (PMSF). To determine protein stability, cells were grown in 100 mL sporulation medium with or without 2% glucose in 500 mL flasks. At the appropriate growth phase, chloramphenicol was added at a final concentration of 150 μg/mL. Then, 25 mL culture was sequentially harvested and washed with 1 mL TBS buffer containing 1 mM PMSF. Cells were disrupted with a French Pressure cell to obtain whole cell extracts. Western blot analysis was performed as previously described (Hata et al., 2001). Monoclonal mouse anti-FLAG M2 antibody (F3165) was purchased from Sigma-Aldrich (Darmstadt, Germany). Polyclonal rabbit anti-SigA antibody was previously described (Ogura, 2016). Monoclonal mouse anti-His tag antibody was purchased from Medical and Biological Laboratories (Nagoya, Japan). These antibodies were diluted (1/1000) in Can Get Signal solution 1 (ToYoBo, Tokyo, Japan). Can Get Signal solution 2 (ToYoBo) was used for POD-conjugated Anti-rabbit/mouse IgG secondary antibody (Roche, Mannheim, Germany). Band intensities were analyzed using Adobe Photoshop version 2.0.

## Results

### Screening for Deficient P*ylxS-lacZ* Expression in Transposon-Mediated Gene Disruptants

**FIGURE 2:**
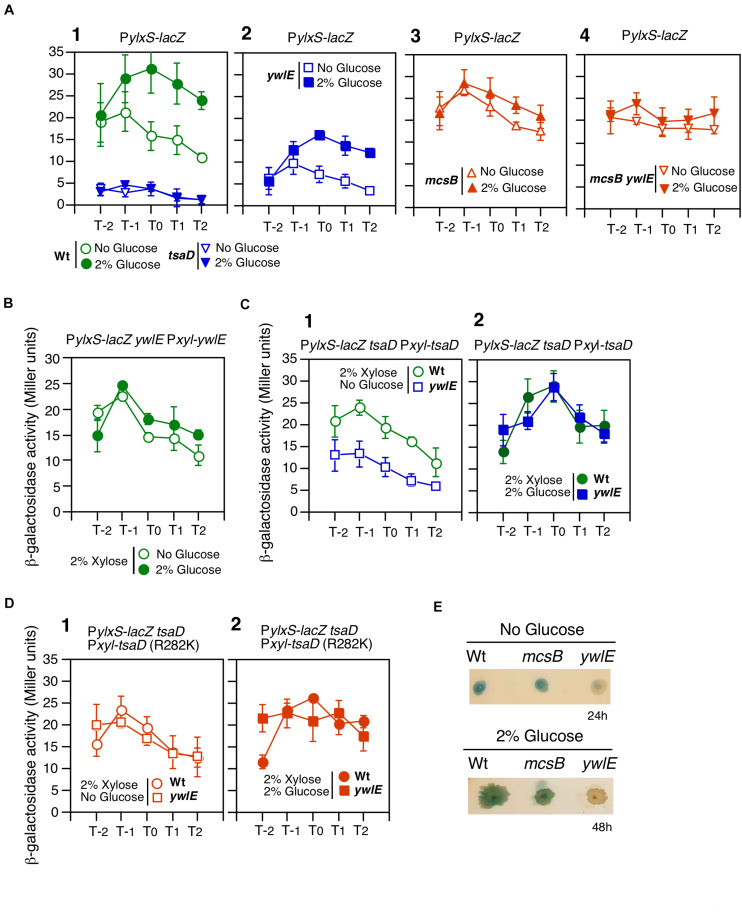
Effects of *ywlE* and *mcsB* disruption and phosphorylated-Arg residue mutation of *tsaD* on P*ylxS-lacZ* expression. **(A–D)** β-Gal activity in sporulation medium. Data represent means and SD from three independent experiments. The *x*-axis represents growth time in hours relative to the end of vegetative growth (T0). The relevant genotype and the presence of glucose or xylose are indicated. The strain lacking *lacZ* showed less than 1 Miller units under the condition with or without glucose. **(A)** P*ylxS-lacZ* expression in mutant strains. 1, OAM741[wild] and OAM882[*tsaD*]; 2, OAM883[*ywlE*]; 3, OAM884[*mcsB*]; 4, OAM945[*ywlE mcsB*]. **(B)** Complementation test of the *ywlE* mutation using OAM885. **(C)** Complementation test of *tsaD* using OAM886[wild] and OAM946[*ywlE*]. **(D)** Complementation test of the *tsaDR282K* mutation using OAM887[Wt] and OAM947[*ywlE*]. **(E)** P*ylxS-lacZ* expression on sporulation medium agar plates. Each strain (OAM741[wild], OAM884[*mcsB*], and OAM883[*ywlE*]) was inoculated onto 1.5% agar sporulation medium plates containing 100 μg/mL X-gal and spectinomycin, and incubated at 37°C. Images were taken at the indicated time.

We previously identified the genes responsible for GI of *sigX*, including *ylxR*, and characterized these genes as a part of a GRS (Figure 1; Ogura and Asai, 2016; Ogura and Kanesaki, 2018; Ogura et al., 2019, 2020). The *ylxR*-containing operon is driven by P*ylxS*, and is composed of the transcription and translation genes, NusA transcription elongation factor and translation initiation factor B (*infB*), respectively (Mondal et al., 2016). This operon is subject to CshA-regulated GI (Ogura and Kanesaki, 2018). To identify additional genes involved in CshA-regulated GI, we screened for transposon (Tn)-insertion mutations that reduced the expression of *ylxS* operon in the presence of glucose. Several candidate genes were obtained from approximately 12,000 colonies. Of these, we identified that *ywlE* encoding an Arg-phosphatase. Tn insertion into the *ywlE* gene reduced P*ylxS-lacZ* expression on solid Schaeffer’s sporulation medium (Figure 2E), although the same strain showed moderately decreased P*ylxS-lacZ* expression compared to wild type in liquid medium irrespective of the presence of glucose (Figures 2A1,2). This difference may be due to different growth conditions. It should be noted that in the *ywlE* disruptant significant GI was observed (see below).

### Involvement of the McsB/YwlE System in P*ylxS* Expression

Next, we performed a complementation test of *ywlE* disruption by artificial overexpression of xylose-inducible *ywlE* at *amyE*. Under xylose and no glucose condition, P*ylxS* expression was higher than that of the *ywlE* disruptant (Figures 2A2,B). This indicated that *ywlE* disruption indeed decreased P*ylxS* expression. Moreover, addition of glucose did not cause GI, suggesting that overproduction of *ywlE* abolished the GI of P*ylxS-lacZ*.

YwlE phosphatase dephosphorylates Arg-phosphorylated proteins, which are generated by McsB kinase. Thus, we examined how *mcsB* disruption affects P*ylxS-lacZ* expression. We observed that in the *mcsB* mutant, basal P*ylxS-lacZ* expression was increased, similar to levels observed under glucose-containing culture, though glucose was not present (Figure 2A3). Thus, *mcsB* disruption abolished GI of this operon. It should be noted that the results in the *mcsB* disruptant were quite similar to those in the *ywlE*-overexpressing strain (Figures 2A3,B). This is reasonable, because *ywlE*-overexpression is essentially equivalent to *mcsB* disruption. When cells had *mcsB* disruption, further disruption of *ywlE* would not affect P*ylxS* expression, because dephosphorylation has no effect if phosphorylation does not occur. This was the case (Figure 2A4). Arg-phosphorylated protein is known to be a target for ClpCP-dependent protein degradation (Trentini et al., 2016). Therefore, GI of P*ylxS-lacZ* expression should be abolished in the *clpC* mutant. We constructed a *clpC*-deficient P*ylxS-lacZ* strain and observed expression with and without glucose. We observed no GI during log-phase but did slight increase of expression with glucose in the early stationary phase due to an unknown reason (Supplementary Figure 2A). Taken together, we concluded that the McsB/YwlE pair is a newly-identified P*ylxS* regulatory factor. Apparently, McsB is involved in GI of P*ylxS* (see section “Discussion”). Moreover, YwlE is required for sufficient expression of P*ylxS*, irrespective of the presence of glucose, under the condition when McsB is functional.

The CshA-regulated GRS regulates *ylxS* and *sigX* promoters. To determine whether McsB/YwlE affects the GRS or specifically P*ylxS*, we examined the effects of the mutations introduced into the *sigX-lacZ* strain. We observed significantly decreased GI of *sigX-lacZ* expression in the *ywlE* disruptant, and only very slightly increased basal expression without glucose in the *mcsB* disruptant (Supplementary Figure 2B). These results show that YwlE affects *sigX* expression similar to P*ylxS*, but to a lesser extent, suggesting that the McsB/YwlE pair is more important for the expression of P*ylxS* than of P*sigX*.

### TsaD as an McsB/YwlE Target Protein in P*ylxS* Expression

We tried to identify the potential McsB/YwlE target in GRS using previous global analyses of Arg-phosphate proteins (Figure 1). From one of the analyses, TsaD was identified as a phosphorylated protein at Arg282 (Trentini et al., 2014). TsaD is a component of the TsaEBD complex, which catalyzes t^6^A-modified tRNA production and plays role in glucose-mediated *sigX* induction via translation of pyruvate dehydrogenase subunits in the presence of glucose (Zhang et al., 2015; Thiaville et al., 2015, 2016; Ogura et al., 2019; Figure 1). In fact, *tsaD* disruption caused severely decreased P*ylxS-lacZ* expression (Figure 2A1). First, we observed that artificial and ectopic expression of xylose-inducible *tsaD* at *amyE* complemented *tsaD* disruption without glucose (compare the expression observed in *tsaD* in Figure 2A1 to wt in Figure 2C1). Next, we observed the decreased P*ylxS-lacZ* expression in the *ywlE* disruptant without glucose (*ywlE* in Figure 2C1). These results demonstrated the negative effect of *ywlE* disruption on P*ylxS* in this *tsaD*-induction system. Under the *tsaD*-overproducing condition with glucose, the similar expression levels of P*ylxS-lacZ* to that in the wild type strain was observed, indicating that *tsaD*-overexpression suppressed the effect of the *ywlE* disruption on P*ylxS* expression (Figure 2C2).

Since the above control experiments worked well, we next examined the possible effect of mutant TsaDR282K on P*ylxS-lacZ* expression. Under the condition where *tsaDR282K* was artificially induced without glucose, P*ylxS-lacZ* expression in the *ywlE* disruptant was similar to that in the strain with functional *ywlE* (Figure 2D1). Thus, the R282K mutation suppressed the negative effect of *ywlE* disruption on P*ylxS* expression. These data indicate that the decreased expression of P*ylxS-lacZ* in the *ywlE* disruptant is dependent on Arg-phosphorylation of TsaD. In the presence of glucose, the mutant strain showed similar phenotypes to the wild-type strain, as expected (Figure 2D2). Moreover, P*ylxS-lacZ* expression was significantly high even without xylose compared to that in the *tsaD* disruptant, probably due to leaky production of TsaD (Supplementary Figure 2C). The results without xylose were similar to those with xylose, indicating that the TsaD protein amounts to sufficiently activate P*ylxS-lacZ* are very small.

### Glucose-Dependent *ywlE* Induction

**FIGURE 3:**
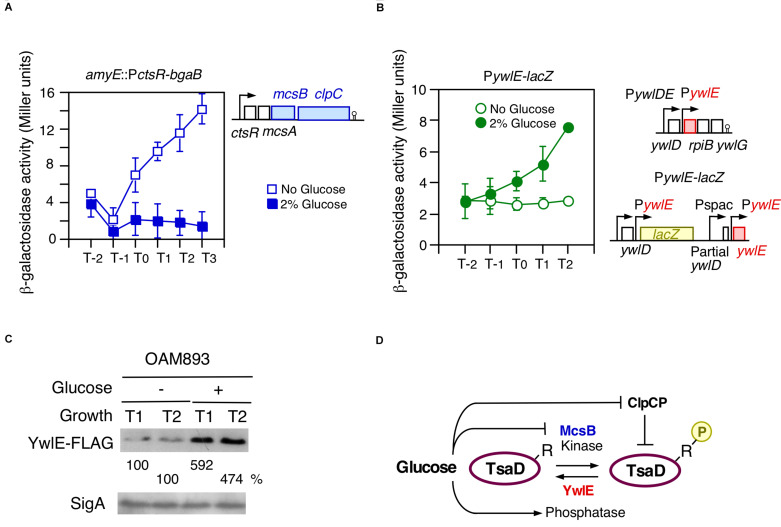
Glucose-induction of *ywlE* and -repression of *mcsB*. **(A,B)** β-Gal activity in sporulation medium using CPRG **(A)** and ONPG **(B)** is shown. Data represent means and SD from three independent experiments. The *x*-axis is the same as in Figure 2. The presence of glucose is indicated. The strain lacking *lacZ/bgaB* showed less than 1 Miller units under the condition with or without glucose. **(A)** P*ctsR-bgaB* expression, OAM891. The chromosomal structure of the *mcsB*-containing operon is shown alongside the panel. **(B)** P*ywlE-lacZ* expression, OAM888. The chromosomal structures of the *ywlE*-containing operon and the region around the P*ywlE-lacZ* fusion are shown alongside the panel. **(C)** Western blot analysis of YwlE-FLAG. The growth phase is indicated in hours relative to the end of vegetative growth (T0) in sporulation medium. Equal protein amounts of whole cell extracts were analyzed in 15% polyacrylamide gels for Western blot using anti-FLAG-tag monoclonal antibody. SigA was used as a control. **(D)** Model of TsaD Arg-phosphorylation control. Arrows and T-bars indicate transcriptional activation or phosphorylation/dephosphorylation, and transcriptional repression or protein degradation, respectively. R, arginine residue to be phosphorylated; circled P, phosphate residue.

Previously, we reported that *ctsR/mcsAB/clpC* operon expression is repressed in glucose-containing sporulation medium (Ishii et al., 2013). Glucose addition also represses ClpC protein expression, which was observed in Western blot analysis (Ishii et al., 2013). The previously observed β-Gal activity was, however, very low, and therefore, we used a more sensitive β-Gal substrate, CPRG. As shown in Figure 3A, glucose addition clearly decreased the P*ctsR* expression. Thus, we examined the possible effect of glucose on *ywlE* expression, because YwlE functionally counteracts McsB. Since *ywlE* expression is driven by two upstream promoters, the *lacZ* reporter gene was inserted into the immediate downstream region of the *ywlE* promoter (Figure 3B). We observed that *ywlE* expression was fourfold induced by glucose after entry into the stationary phase (Figure 3B). P*ywlE-lacZ* showed a glucose-dependent response at more than 0.5% glucose, which was also induced by glycerol but not acetate or succinate (data not shown). These carbon source reactivities were similar to those of P*ylxS-lacZ* (Ogura and Kanesaki, 2018). To determine which of the two promoters are responsible for GI of *ywlE*, we constructed ectopic *lacZ*-fusions with the 0.4 and 1.0 Kb upstream regions of *ywlE* at *amyE*. The short and long fusions contain P*ywlE* and both promoters, respectively. The two fusion expression was only 3- to 2.5-fold induced by glucose (Supplementary Figure 2D). These results indicated that P*ywlE* is responsible for GI and that GI of *ywlE* was fully observed only in the original chromosome region. This may be due to chromosomal position effect (Bryant et al., 2014).

The GI of *ywlE* was also observed at the protein level as expected (Figure 3C). Notably, *ywlE*-FLAG is functionally equivalent to the wild type protein because in the tag-carrying strain, similar GI and basal expression of P*ylxS-lacZ* were observed (left, Supplementary Figure 2E). These findings strongly suggested that the TsaD phosphorylation state is glucose-dependent, leading to altered protein levels from ClpCP-dependent degradation of Arg-phosphorylated proteins (Figure 3D).

### Glucose-Mediated Control of TsaD Degradation

**FIGURE 4:**
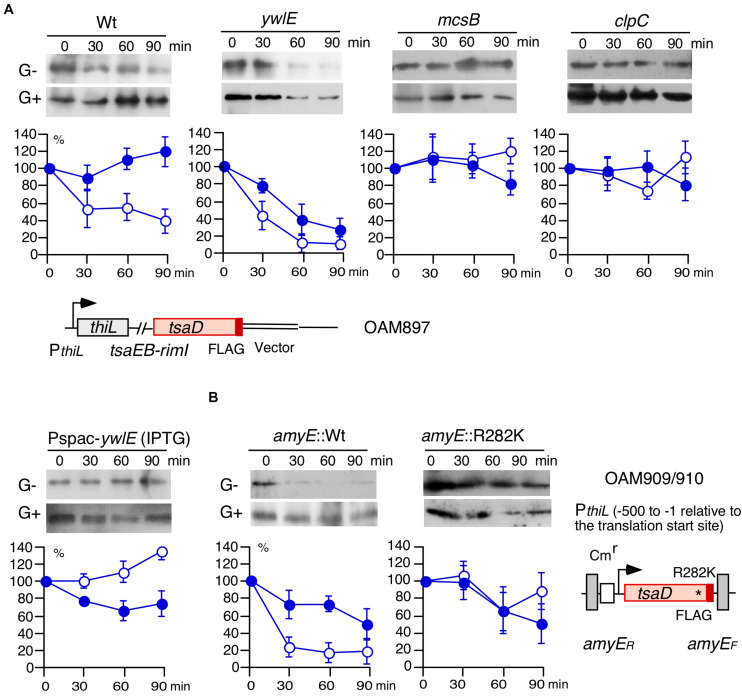
Western blot analysis of TsaD-FLAG. Equal protein amounts of whole cell extracts were analyzed in 12.5% polyacrylamide gels for Western blots using anti-FLAG-tag monoclonal antibody. The chromosomal structures of OAM897 and OAM 909/910 are shown. Boxes and bent arrows show open-reading frames and promoters, respectively. The promoter region (−500/−1 relative to the translation start site) has full promoter activity (Ogura et al., 2019). **(A,B)** Protein stability analysis. Band intensities are shown in the graphs. After inhibiting protein synthesis, more than 100% of Arg-phosphorylated TsaD was sometimes observed. It is likely that apparent ratio of TsaD, if not degraded, to the total protein amounts increased due to other protein degradation systems. Time indicates culture sampling interval after chloramphenicol addition, which was added at T1 in sporulation medium culture. G+ and G− indicate the presence or absence of 2% glucose. Closed and open symbols indicate results from the medium containing glucose or no glucose, respectively. Means and SD (error bars) are shown from three to five biologically-independent samples. A, OAM897[wild], OAM898[*ywlE*], OAM899[*mcsB*], OAM900[*clpC*], and OAM901[Pspac-*ywlE*]. For OAM901, 1 mM IPTG (final concentration) was added. B, OAM909[wild] and OAM910[R282K mutant]. * indicates the introduced nucleotide change.

Since Arg-phosphorylated TsaD may be subjected to ClpCP-dependent degradation, we examined TsaD stability using Western blot analysis. The FLAG-tagged TsaD is functionally equivalent to the wild type protein, because similar GI and basal expression of P*ylxS-lacZ* was observed to that in the wild-type strain (right, Supplementary Figure 2E). To determine protein stability, which we defined as protein degradation rate after inhibiting protein synthesis, chloramphenicol was added to the culture medium. TsaD-FLAG protein was quantified in sequentially-sampled cells. First, we examined wild type strain without glucose and observed fast protein degradation, however, in cells grown with glucose, TsaD-FLAG was stabilized (left, Figure 4A). In the *ywlE* disruptant, TsaD-FLAG was more unstable compared to the wild-type strain irrespective of the presence of glucose (mid-left, Figure 4A). The observation of glucose-mediated stabilization even in the *ywlE* disruptant is reasoned by catabolite repression of *mcsB* and *clpC.* Based on these results, it is possible that *mcsB* or *clpC* disruption stabilizes TsaD-FLAG. Similarly, *ywlE* overproduction, which should be equivalent to *mcsB* disruptant, would stabilize TsaD-FLAG. Thus, we examined protein stability in these strains and observed the expected TsaD-FLAG stabilization irrespective of the presence of glucose (Figure 4A). Next, to confirm the significance of R282, we constructed an ectopic TsaD-FLAG expression system driven by its own promoter at *amyE* (Figure 4B). Subsequently, the Arg residue to be phosphorylated was changed to Lys in the TsaD protein at *amyE* (TsaDR282K). Using the expression system, we observed similar protein degradation profiles of wild-type protein as in the cases of the strain bearing TsaD-FLAG in its original location (left, Figures 4A,B). As expected, the mutant TsaDR282K-FLAG protein was significantly stabilized in the absence of glucose compared to the wild type (Figure 4B). Similar degradation rates were observed for wild and mutant TsaD with glucose. These were consistent with the observations for the wild and *mcsB* strains bearing TsaD-FLAG in original location. These results indicate that the McsB/YwlE system, including ClpCP, is involved in the control of TsaD stability. These results are consistent with the results shown in Figure 2, and strongly suggest that control of TsaD stability through Arg-phosphorylation regulates P*ylxS-lacZ* expression.

### Glucose-Mediated PdhD-His Protein Stabilization and Effect of *ywlE/mcsB* Mutation on Cell Growth

**FIGURE 5:**
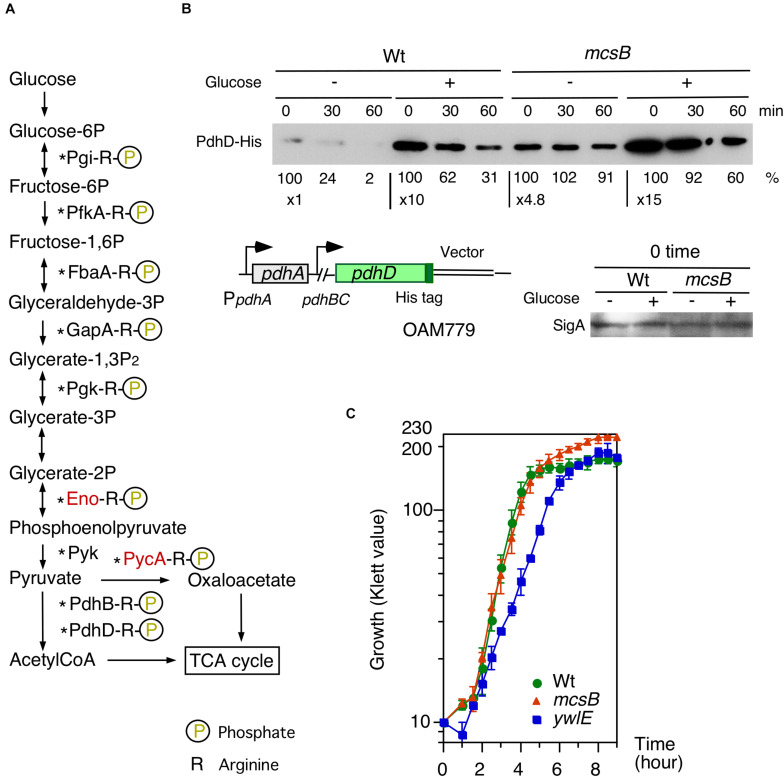
Effects of glucose on PdhD-His stability and cell growth profiles in *mcsB* and *ywlE* disruptants. **(A)** Glycolytic pathways and enzymes detected in Arg-phosphorylated forms. Enzymes in red letters are likely to be regulated by ClpCP-dependent degradation (Gerth et al., 2008). Asterisks show the enzymes that have been reported to be acetylated (Kosono et al., 2015; Carabetta et al., 2016). **(B)** PdhD-His western analysis. Cells were grown in sporulation medium with and without 2% glucose. Equal protein amounts of whole cell extracts were analyzed in 12.5% polyacrylamide gel for Western blots using anti-His-tag monoclonal antibody. Time indicates sampling interval after chloramphenicol addition, which was added at T1. Band intensities are indicated below the panels. The chromosomal structure of OAM779 is shown. Boxes and bent arrows show open-reading frames and promoters, respectively. OAM779 [Wt] and OAM908 [*mcsB*]. As a control, SigA is shown for time 0 samples. **(C)** Cell growth profiles of each mutant. Overnight culture grown in A3 medium was inoculated to 4 mL semisynthetic MC (modified competence) medium in an L-tube. Growth was monitored with a Klett calorimeter (Thermo Fisher Scientific, Waltham, MA, United States). Means and SD from three independent experiments are shown. 168[wild], OAM879[*mcsB*], and OAM881[*ywlE*].

Many glycolytic and TCA-cycle enzymes, including PdhD, which is involved in P*ylxS* regulation, were previously identified in the analysis of Arg-phosphorylated proteins (Elsholz et al., 2012; Schmidt et al., 2014; Trentini et al., 2014; Figures 1, 5A). We observed that PdhD-His was degraded after quenching protein synthesis (Figure 5B). Moreover, in the *mcsB* disruptant, PdhD-His was more stabilized than the case for the wild-type, strongly suggesting that this degradation is mediated by PdhD Arg-phosphorylation. Based on these observations and the model shown in Figure 3D, we expected that glucose addition to the culture medium would result in lower protein degradation because of glucose-mediated *ywlE* induction and *mcsB* repression. Our results confirmed this notion (Figure 5B). Notably, glucose addition results in an increase of *pdhABCD* operon transcription (Blencke et al., 2003). This effect would reflect enhanced protein expression by glucose. To further examine the Arg-phosphorylation effects on PdhD protein stability, we constructed an ectopic PdhD-His expression system driven by the *pdhABCD* operon promoter at *amyE*. The PdhD-His from this strain, however, was highly unstable and addition of glucose did not significantly stabilize the protein (data not shown). Thus, we did not perform this mutational analysis. Based on these results, it was concluded that the protein degradation of PdhD is mediated by Arg-phosphorylation.

According to the experimental results on glucose-mediated stability control of PdhD, we expected that in glucose-containing medium, YwlE/McsB may be involved in cell growth. To test this, we used semi-synthetic medium, because more stable cell growth profiles are obtained compared to complex medium containing natural components. The semi-synthetic MC (modified competence) medium contains 2% glucose, 0.1% citrate, 0.2% glutamate, 0.1% casamino acids, tryptophan, salts, and minerals (Kunst et al., 1994). It should be noted that in this medium the P*ctsR-bgaB* and P*ywlE-lacZ* expression was similar to those in glucose-added sporulation medium (Supplementary Figure 2F). The *ywlE* disruptant showed delayed cell growth in the log-phase, but overall cell mass was similar to the wild-type (Figure 5C). However, while the *mcsB* disruptant showed a similar cell growth profile in the log-phase, the final cell mass was significantly larger and about 40% increased. These results show that McsB and YwlE affect cell growth in MC medium.

## Discussion

In this study, we identified the McsB/YwlE system controlling Arg-phosphorylation of target proteins in GRS, particularly as a regulatory factor for TsaD. Since Arg-phosphorylated proteins are degraded by ClpCP, the McsB/YwlE system regulates the fate of such proteins. In fact, we observed changes of degradation rates of TsaD after mutations to the McsB/YwlE system or substitution of the Arg-to-Lys mutation in TsaD. TsaD would function via efficient translation through modification of tRNA decoding ANN codons. Thus, glucose-mediated TsaD enhancement may have global effects on cellular physiology, because many proteins have ANN codons.

**FIGURE 6:**
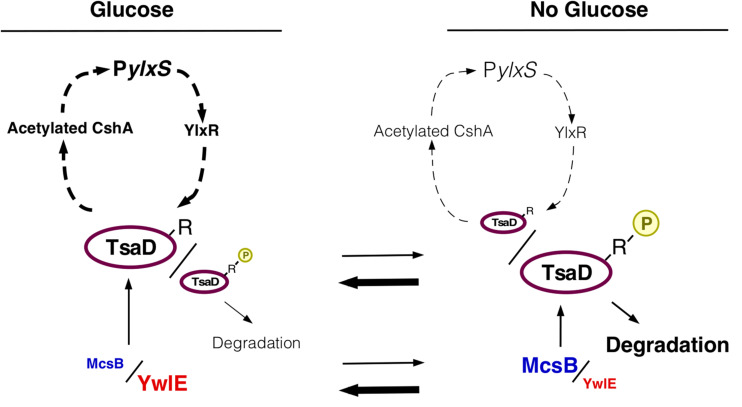
Working hypothesis of glucose-mediated P*ylxS* regulation through TsaD in GRS. Description of figures is the same as in Figures 1, 3D. The arrowheads with dotted line indicate indirect positive regulation (see Figure 1).

Overproduction of *tsaD* resulted in abolishment of GI of P*ylxS-lacZ*, whereas simultaneous overproduction of *tsaD* and *ywlE* disruption, which decreased protein stability of TsaD, lowered P*ylxS* expression in the absence of glucose and rescued GI. This GI would be attributed to catabolite repression of *mcsB* because YwlE is abcent. These observations indicated that glucose-mediated increase from the low levels of cellular amount of TsaD is critical for GI of P*ylxS*. The fact that the overproduction of more stable TsaDR282K in the strain with *ywlE* disruption did not show GI of P*ylxS* is consistent with the above notion. In the *tsaD*-overexpressing strain, there was no further increase in P*ylxS* expression from that in the wild type strain even with glucose, suggesting that these levels of TsaD might also exceed the saturation levels required for P*ylxS* expression. Otherwise, too much amount of TsaD might inhibit further enhancement of P*ylxS* expression. Disruption of *mcsB* or overproduction of *ywlE*, which is equivalent to *mcsB* disruption, also resulted in abolishment of GI of P*ylxS-lacZ.* Since the *mcsB* disruptant lacks the glucose target and requirement of another target, *ywlE*, is canceled because of absence of McsB, it is reasonable that addition of glucose has no effect on P*ylxS* expression. These results support the notion that P*ylxS-lacZ* induction is caused by glucose-mediated control of Arg-phosphorylation state of TsaD. On the contrary, YwlE is a positive factor for P*ylxS* expression, when McsB is present. Collectively, these results indicate a central role for McsB/YwlE in GI of P*ylxS-lacZ* (Figure 6). When glucose was added, TsaD levels increased, probably leading to efficient translation of the *pdhABCD* operon. In addition, glucose increased *pdhABCD* transcription and stabilized PdhD. Perhaps PdhB is also stabilized through decreased Arg-phosphorylation. Then, enhanced PDH levels would stably supply acetyl-CoA as a source of acetylated CshA, which is a positive regulator for P*ylxS*. Then P*ylxS*-driven *ylxR* activates *tsaD*-containing operon expression (Figure 6).

Signals controlling the McsB/YwlE system occur in two environmental states–heat-shock and oxidative stress (Krueger et al., 2001; Fuhrmann et al., 2016). Heat-shock induces the *ctsR/mcsAB/clpC* operon through an intrinsic CtsR thermosensor function. McsA and McsB are required for CtsR degradation. Oxidative stress is sensed by cysteine residue of YwlE, leading to YwlE inactivation (Fuhrmann et al., 2016). The global analyses of Arg-phosphorylated proteins revealed many genes not involved in heat-shock and oxidative stress responses. Indeed, presence of several other unknown signals for the McsB/YwlE system has been pointed out (Suskiewicz and Clausen, 2016). Our study raised the possibility that glucose availability regulates the McsB/YwlE system, because glucose induced P*ywlE-lacZ* and repressed P*ctsR-bgaB*. The expression of P*ctsR-bgaB* is activated by Spx and repressed by CtsR (Elsholz et al., 2017; Rojas-Tapias and Helmann, 2019). We note that in the *ctsR-*inactivation background, elevated P*ctsR-bgaB* expression was still repressed by glucose (Ishii et al., 2013). In the *spx* inactivation background, P*ctsR-bgaB* expression was further repressed by glucose (Ogura M, unpublished results).

The McsB/YwlE system has profound effects on gene transcription, including the ComK-regulon (Elsholz et al., 2012). Moreover, genome-wide analysis of Arg-phosphorylated proteins indicated DegU, a transcription factor controlling multiple physiological phenomena including biofilm formation, genetic competence, motility, and exoprotease production, is Arg-phosphorylated (Kobayashi, 2007; Tsukahara and Ogura, 2008; Ogura and Tsukahara, 2010; Chan et al., 2014; Schmidt et al., 2014; Trentini et al., 2014, 2016; Ogura, 2016). We performed *in vitro* degradation assays using Asp-phosphorylated DegU and showed ClpCP-dependent DegU-P degradation (Ogura and Tsukahara, 2010). In this system, the role of McsB/YwlE should be clarified by future study. Recent studies revealed that the McsB/YwlE system plays critical roles for spore germination (Zhou et al., 2019). Immediately after receiving trigger nutrients, activated YwlE dephosphorylates Arg-phosphorylated SigA and Tig, important factors for transcription and translation, respectively. As a result, the *ywlE* disruptant showed delayed germination. This is a distinct physiological phenotype of the *ywlE* disruptant.

In the glucose-based semi-synthetic medium, the *ywlE* disruptant showed decreased growth rate (Figure 5C). This raised the possibility that Arg-phosphorylated glycolytic enzyme dephosphorylation plays a role in normal cell growth. Indeed, degradation of Eno and Pyc by ClpCP have been identified in 2D gel analysis of the *B. subtilis* proteome after culture in minimal medium with glutamate and without citrate (Gerth et al., 2008). Moreover, these results are consistent with previous observations that Eno and Pyc are Arg-phosphorylated proteins (Schmidt et al., 2014; Trentini et al., 2014). However, the study addressing the role of Arg-phosphorylation of GapA revealed that GapA was stable after change of glucose to malate as carbon source (Gerth et al., 2017). The recent study showed that pyruvate kinase significantly affects Z-ring formation and showed that pyruvate is a key metabolite that coordinates bacterial growth and cell division (Monahan et al., 2014). These metabolic activities, which may be regulated by YwlE, would be related to cell growth. Moreover, many glycolytic enzymes are acetylated, such as Pgi, GapA, Pgk, PdhD, and PycA (Kosono et al., 2015). These proteins have been reported to be Arg-phosphorylated, perhaps leading to degradation (Schmidt et al., 2014; Trentini et al., 2014). In *B. subtilis*, acetylated Eno is inhibited and FbaA and Pyk are likely to be acetylated at critical lysine residues for enzyme activities (Nakayasu et al., 2017). Considering glucose-mediated *ywlE* induction, glucose has positive (dephosphorylation of Arg-phosphorylated enzyme leading to protein stabilization, which is the case for PdhD) and negative effects (acetylation and inhibition) on glycolytic enzymes through these two protein modification systems. Perhaps the overall balance between both effects might be likely to be positive, because we observed that in the presence of glucose, wild-type cells showed significantly higher cell mass at the early stationary phase compared to cells without glucose (Ogura et al., 2019). Moreover, a recent study showed that wild type, *mcsB*, and *ywlE* strain growth was unchanged in LB medium, which hardly contains glucose (Zhou et al., 2019), confirming our observation that growth changes are dependent on glucose. The *mcsB* disruptant showed increased cell mass at the early stationary phase. In the *mcsB* disruptant, glycolytic enzymes are not Arg-phosphorylated, suggesting that glycolytic enzyme levels should be high, leading to more available energy. This would result in increased cell mass. Together, the cell growth phenotypes of the *mcsB* and *ywlE* disruptants probably involve Arg-phosphorylation-mediated protein degradation of glycolytic enzymes, such as PdhD. Arg-phosphorylation-mediated and ClpCP-dependent *in vivo* protein degradation of specific proteins under normal conditions is likely, but thus far remain unidentified (Schmidt et al., 2014).

Glucose has profound effects, including CcpA-mediated repression and activation of more than a hundred genes. Further, glucose likely activates and inhibits several unidentified transcription factors (for example, the transcription factor responsible for glucose-mediated *pdhABCD* induction is not known), and participates in transcriptome alterations (Blencke et al., 2003; Deutscher, 2008; Fujita, 2009). CcpA is indirectly involved in catabolite repression of *ctsR*/*mcsAB/clpC* and the previous transcriptome analyses revealed that CcpA is involved in regulation of *ctsR/clpC* expression in a synthetic medium (Moreno et al., 2001; Ishii et al., 2013). Our preliminary RNA-seq analysis to assess the effect of glucose on the transcriptome (cells grown in sporulation medium at T1) showed decreases of mRNA levels of *ctsR, mcsA*, *mcsB* and *clpC* to 44, 26, 30, and 31% of respective levels in the wild type strain (Ogura and Kanesaki, unpublished results). These are consistent with catabolite repression of P*ctsR*. Taken together, our findings indicate that (1) TsaD is a target protein of McsB/YwlE, (2) glucose induces *ywlE* and represses *mcsB/clpC*, and (3) McsB/YwlE affects cell growth in glucose-containing semi-synthetic medium. The overall results provide profound insights on understanding *B. subtilis* cell physiology responses to environmental cues, including glucose.

## Data Availability Statement

All datasets presented in this study are included in the article/Supplementary Material.

## Author Contributions

MO performed the experiments and wrote the manuscript.

## Conflict of Interest

The author declares that the research was conducted in the absence of any commercial or financial relationships that could be construed as a potential conflict of interest.
