# Supplementary material for: Glucose-Mediated Protein Arginine Phosphorylation/Dephosphorylation Regulates ylxR Encoding Nucleoid-Associated Protein and Cell Growth in Bacillus subtilis
Source: Front Microbiol. 2020 Sep 25;11:590828. doi: 10.3389/fmicb.2020.590828 (PMC7546277; doi:10.3389/fmicb.2020.590828)
Supplement: Supplementary file 1 [file Data_Sheet_1.PDF]

**Fig. S1. Schematic representation of the construction of *amyE*::P*thiL-tsaD*.** Primer pairs for each PCR are shown below the scheme. Boxes and bent arrows show open-reading frames and promoters, respectively.

**Fig. S2. P*ylxS-lacZ*, P*sigX-lacZ*, P*ywlE-lacZ*, or P*ctsR-bgaB* expression in various mutants and media.** β-Gal activity. Data represent means and standard deviations from three independent experiments. The *x*-axis represents the growth time in hours relative to the end of vegetative growth (T0). The relevant genotype, the presence of glucose, or the presence of xylose are indicated below the panel. **A-E**, sporulation medium and **F,** MC medium. **A.** Expression of P*ylxS-lacZ* in the *clpC* mutant. OAM904 [*trpC2 thrC*::P*ylxS-lacZ* (-284/+77 relative to the transcription start site, $Sp^r$) *clpC* ($Tc^r$)]. **B.** Expression of *sigX-lacZ*. BSU43 [*trpC2 amyE*::*sigX-lacZ* (-43 to +262, relative to the transcription start site, $Cm^r$)] (Kosono et al., FEMS Microbiol Lett, 2004, 232:93). OAM902 and OAM903 are derivatives of BSU43 with *ywlE*::$Km^r$ and *mcsB*::$Em^r$-$Tc^r$, respectively. **C.** Complementation test of the *tsaD* mutation using OAM886, OAM946, OAM887, and OAM947. The same experiments in Fig. 2C and 2D without xylose are shown. **D.** Expression of *amyE*::P*ywlDE-lacZ* and *amyE*::P*ywlE-lacZ,* which were made by transformation of pIS-PywlDE and pIS-PywlE to the 168 chromosome, respectively. To construct pIS−PywlDE and pIS−PywlE, PCR products were amplified using the oligonucleotides pairs pIS-PywlDE-F(E)/pMut-PywlE-R(B) and pIS-PywlE-F(E)/pMut-PywlE-R(B), digested with *Eco*RI/*Bam*HI, and cloned into pIS248 treated with the same enzymes (Ogura et al, 2003, Mol Microbiol 49:1685). OAM948 [*trpC2 amyE*::P*ywlDE-lacZ* (-1031/+1 relative to the translation start site of *ywlE*, $Cm^r$)] and OAM949 [*trpC2 amyE*::P*ywlE-lacZ* (-399/+1 relative to the translation start site of *ywlE*, $Cm^r$)]. **E.** Expression of P*ylxS-lacZ* with *ywlE-flag* (left) and *tsaD-flag* (right). OAM906 [*trpC2 thrC*::P*ylxS-lacZ* (-284/+77, $Sp^r$) *ywlE-flag* ($Cm^r$)] and OAM907 [*trpC2 thrC*::P*ylxS-lacZ* (-284/+77, $Sp^r$) *tsaD-flag* ($Cm^r$)]. **F.** Expression of P*ywlE-lacZ* (OAM888) and P*ctsR-bgaB* (P*ctsR-bgaB*).

Table S1. Oligonucleotides used for this study.

| Name | Sequence | Product/use |
|---|---|---|
| 695 | 5'-GCTTGTAAATTCTATCATAATTG-3' | Transposon mutagenesis |
| 696 | 5'-AGGGAATCATTTGAAGGTTGG-3' | Transposon mutagenesis |
| pMut-PywlE-F(H) | 5'-TCAAAGCTTGTTCTTGGGGTTCAAATGC-3' | pMutin-PywlE |
| pMut-PywlE-R(B) | 5'-TAGGGATCCGTCAGTCACCCCTTATTTTTCTC-3' | pMutin-PywlE, pIS-PywlE, pIS-PywlDE |
| pIS-PywlE-F(E) | 5'-TCAGAATTCGCTGCTTTTTGTTCTTGG-3' | pIS-PywlE |
| pIS-PywlDE-F(E) | 5'-TCAGAATTCTCAGAATTCGCCGACAGCGCCTCTTG-3' | pIS-PywlDE |
| pX-ywlE-Spe | 5'-AAACTAGTGAGAAAAATAAGGGGTGACTGA-3' | pX-ywlE |
| pX-ywlE-Bam | 5'-ATGGGATCCTTATCTACGGTCTTTTTTCAGCTG-3' | pX-ywlE |
| pX-gcp-Spe | 5'-AAACTAGTAATTATGTGGGTGACGATAAATG-3' | pX-tsaD, pX-tsaD-m |
| pX-gcp-Bam | 5'-ATGGGATCCTTATCTCGTGAGACTTTGATAAG-3' | pX-tsaD, pX-tsaD-m |
| tsaD-M1 | 5'-GCAAACAGAGGCCTCAaAGCTGCATTAGAAAA-3' | pX-tsaD-m, OAM910 |
| tsaD-M2 | 5'-TTTTCTAATGCAGCTTTGAGGCCTCTGTTTGC-3' | pX-tsaD-m, OAM910 |
| YwlE-FF | 5'-GCATCATGGCGTTTGCTTTAG-3' | *ywlE*::$Tc^r$ |
| YwlE-(Tc)-FR | 5'-GCTGTTCATATCGACCAATGTGTTTTTCAAACAGCGCTTC-3' | *ywlE*::$Tc^r$ |
| YwlE-(Tc)-RF | 5'-TTTTTTTATAACAGGAATTCCTGGCAAAACAGCTGAAAAAAG-3' | *ywlE*::$Tc^r$ |
| YwlE-RR | 5'-TTGTTTCAATTCCGCTTGGTCCT-3' | *ywlE*::$Tc^r$ |
| TC-F | 5'-GGTCGATATGAACAGCTTATTTAC-3' | *ywlE*::$Tc^r$ |
| TC-R | 5'-GAATTCCTGTTATAAAAAAAGGATCAA-3' | *ywlE*::$Tc^r$ |
| Pflag-tsaD-F-H | 5'-TCAAAGCTTGCCATATCCGGGTGGAC-3' | ptsaD-flag |
| Pflag-tsaD-R-Xb | 5'-TAGTCTAGATCTCGTGAGACTTTGATAAGAA-3' | ptsaD-flag |
| Pflag-ywlE-F-H | 5'-TCAAAGCTTGTCTGTACTGGAAATACGTG-3' | pywlE-flag |
| Pflag-ywlE-R-Xb | 5'-TAGTCTAGATCTACGGTCTTTTTTCAGCTGTT-3' | pywlE-flag |
| amyE-RR | 5'-TCAATGGGGAAGAGAACCGCTTAAGCCCG-3' | OAM909, OAM910, OAM911, OAM912 |
| amyE-FF | 5'-GTTAACAAAATTCTCCAGTCTTCACATCGG-3' | OAM909, OAM910, OAM911, OAM912 |
| Cat-R/gcp-E | 5’-GGGTGTGAATTCAATGCACCCATTAGTTCAACAAACG-3' | OAM909, OAM910 |
| pDG1729-gcp-E | 5'-ATTGAATTCACACCCGTTCCCATACCGAACA-3' | OAM909, OAM910 |
| pDG1729-gcp-B | 5'-ATCGGATCCGAACCAAAACCTCCTTTCAACC-3' | OAM909, OAM910 |
| Gcp-B/tsaD-F | 5'-TGGTTCGGATCCGATAATTATGTGGGTGACGATAAATG-3' | OAM909, OAM910 |
| Px-FLAG-R-Bg | 5'-TTCAGATCTTCACTACTTGTCATCGTCATCC-3' | OAM909, OAM910 |
| amyE-FR-flag | 5'-GTAGTGAAGATCTGAACGATCAGACCAGTTTTTAATTTG-3' | OAM909, OAM910 |

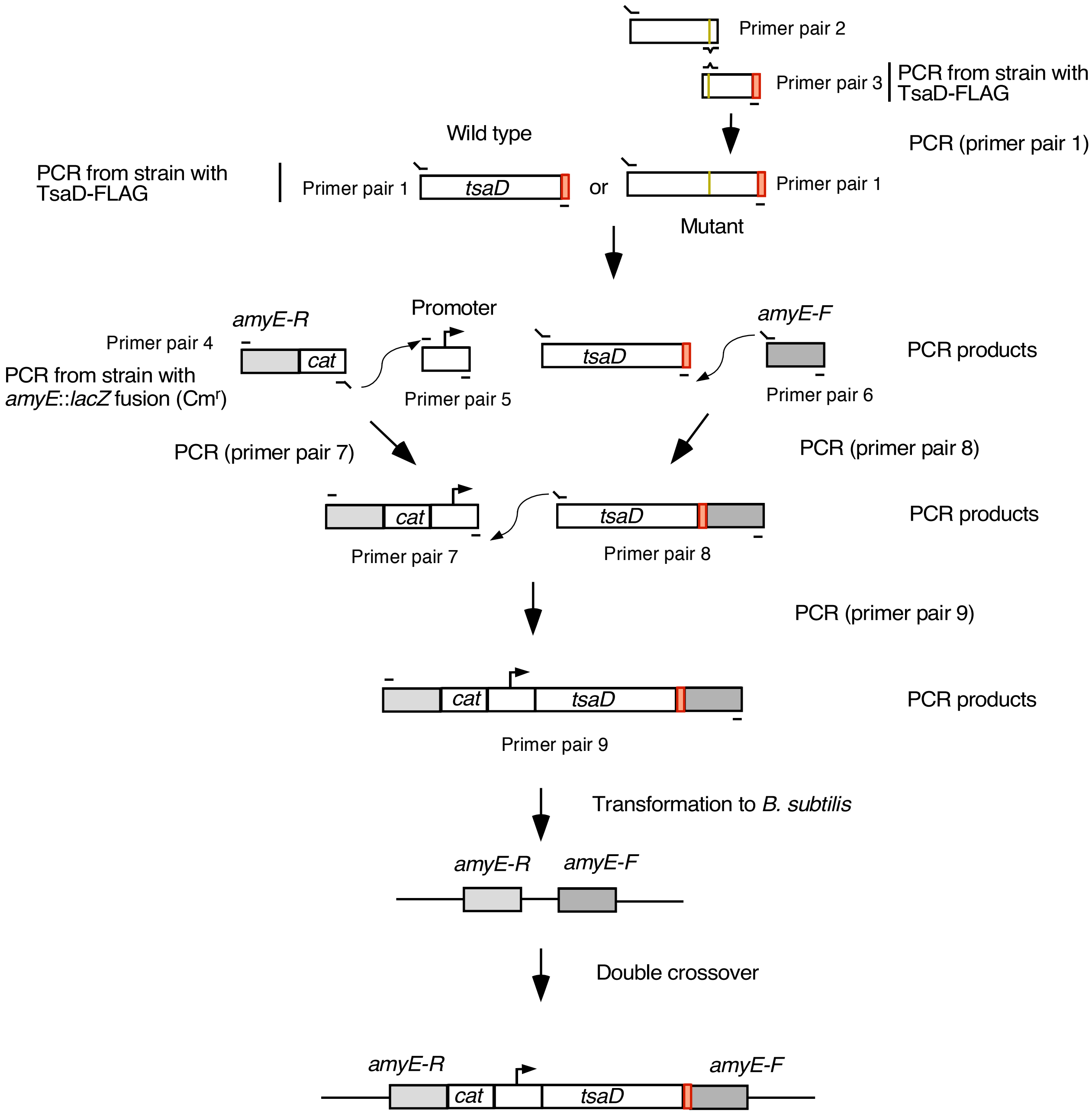


| For TsaD-FLAG |
|---|
| Primer pair 1 : Gcp-B/tsaD-F, Px-FLAG-R-Bg |
| Primer pair 2 : Gcp-B/tsaD-F, tsaD-M2 |
| Primer pair 3 : tsaD-M1, Px-FLAG-R-Bg |
| Primer pair 4 : amyE-RR, Cat-R/gcp-E |
| Primer pair 5 : pDG1729-gcp-E, pDG1729-gcp-B |
| Primer pair 6 : amyE-FR-flag, amyE-FF |
| Primer pair 7 : amyE-RR, pDG1729-gcp-B |
| Primer pair 8 : Gcp-B/tsaD-F, amyE-FF |
| Primer pair 9 : amyE-RR, amyE-FF |

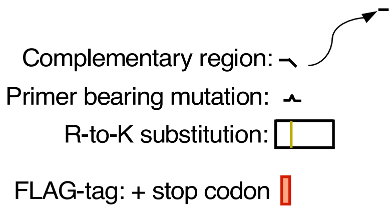


Fig. S1

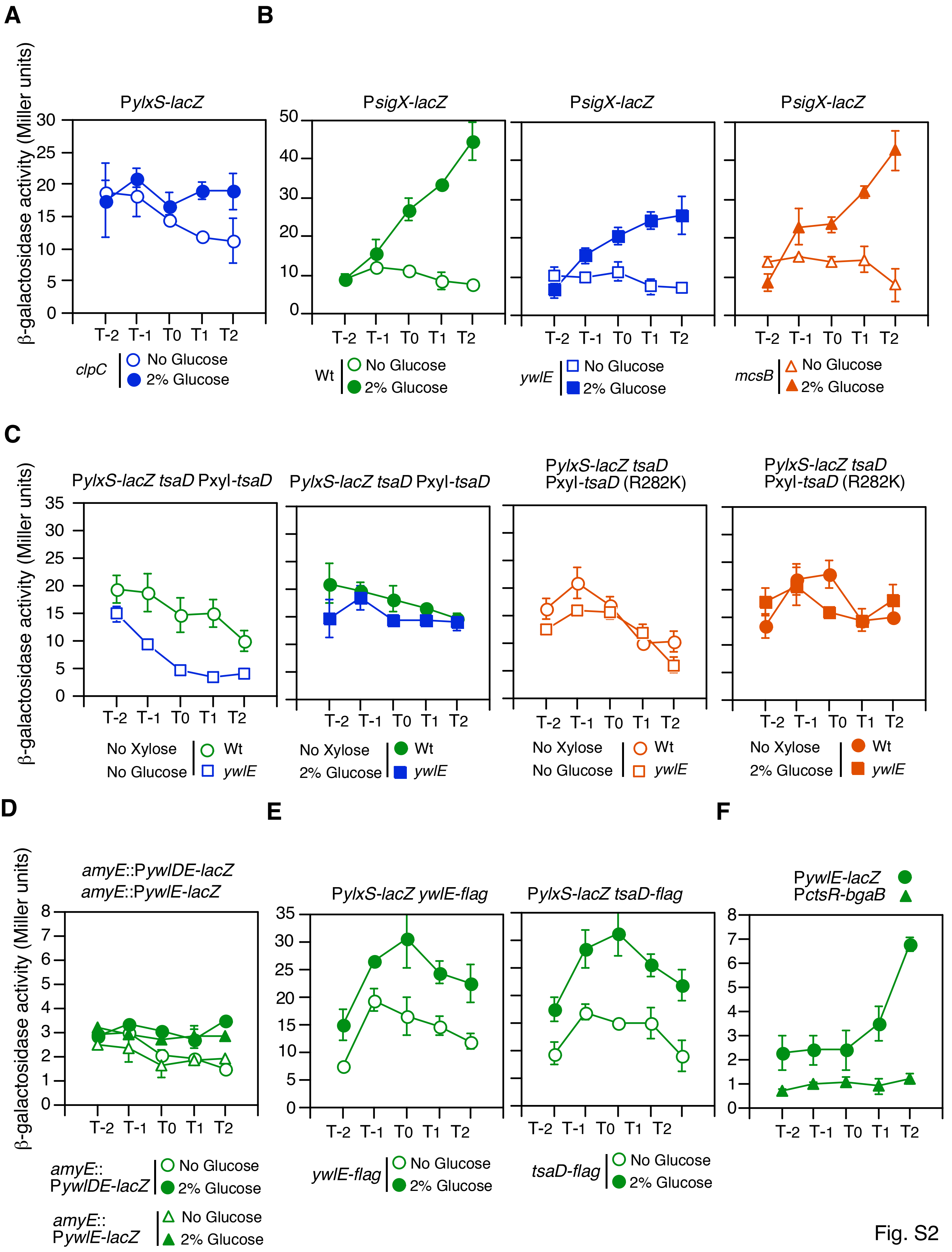
A
B
C
D
E
F
β-galactosidase activity (Miller units)
PylxS-lacZ
PsigX-lacZ
T-2 T-1 T0 T1 T2
clpC
No Glucose
2% Glucose
Wt
ywlE
mcsB
PylxS-lacZ tsaD Pxyl-tsaD
PylxS-lacZ tsaD Pxyl-tsaD (R282K)
No Xylose
No Glucose
2% Glucose
amyE::PywlDE-lacZ
amyE::PywlE-lacZ
PylxS-lacZ ywlE-flag
PylxS-lacZ tsaD-flag
ywlE-flag
tsaD-flag
PywlE-lacZ
PctsR-bgaB

Fig. S2
